# Effect of green tea and lycopene on the insulin-like growth factor system: the ProDiet randomized controlled trial

**DOI:** 10.1097/CEJ.0000000000000502

**Published:** 2018-03-15

**Authors:** Kalina M. Biernacka, Jeff M.P. Holly, Richard M. Martin, Aleksandra Frankow, Caroline J. Bull, Freddie C. Hamdy, Jenny L. Donovan, David E. Neal, Chris Metcalfe, Athene Lane

**Affiliations:** a IGFs & Metabolic Endocrinology Group, Translational Health Sciences, Bristol Medical School, Southmead Hospital; b National Institute for Health Research (NIHR) Biomedical Research Centre at University Hospitals Bristol NHS Foundation Trust and the University of Bristol; c Population Health Sciences, Bristol Medical School, University of Bristol; d Bristol Randomised Trials Collaboration, School of Social and Community Medicine, Bristol; e Nuffield Department of Surgery, University of Oxford; f Faculty of Medical Science, John Radcliffe Hospital, Oxford; g Department of Oncology, Addenbrooke’s Hospital, University of Cambridge; h Cancer Research UK Cambridge Research Institute, Li Ka Shing Centre, Cambridge, UK

**Keywords:** diet, green tea, insulin-like growth factor binding protein-3, insulin-like growth factor-I, lycopene, prostate cancer

## Abstract

Whether prostate cancer (PCa) may be preventable by dietary interventions can be assessed in randomized trials using intermediate biomarkers of cancer risk or progression. We investigated whether lycopene or green tea modify circulating insulin-like growth factor (IGF) peptides in men at increased risk of PCa. Participants (aged 50–69 years) in one centre in the UK wide PCa testing and treatment trial (ProtecT) with prostate specific antigen between 2.0 and 2.95 ng/ml or negative biopsies, were randomized to daily lycopene (*n* = 44 assigned 15 mg capsules/day; 44 assigned a lycopene-rich diet; 45 assigned placebo) and green tea (*n* = 45 assigned 600 mg/day epigallocatechin gallate; 45 assigned green tea drink; 43 assigned placebo) for 6 months. The interventions significantly elevated the primary outcomes, serum epigallocatechin gallate and lycopene at 6 months of follow-up. We report here an exploratory analysis in which serum IGF-I, IGF-II, IGF binding protein (BP)-2 and IGFBP-3 were measured at baseline and 6 months of postintervention. A total of 133 men were randomized (34% of eligible men approached) and 130 had follow-up IGF peptides (98%). In intention-to-treat analyses, there was only weak evidence that lycopene or green tea influenced some aspects of serum IGF-I, IGF-II, IGFBP-2 or IGFBP-3. In men randomized to lycopene supplements, IGFBP-2 was nonsignificantly (50.9 ng/ml; 95% confidence interval: −51.2–152.9, *P* = 0.3) higher in comparison to placebo, whereas in men randomized to green tea supplements, IGFBP-3 was nonsignificantly (205.2 ng/ml; 95% confidence interval: −583.3–172.9, *P* = 0.3) lower than with placebo. In this small, pilot randomized controlled trial, there was little evidence that lycopene or green tea interventions influenced serum levels of IGF-I, IGF-II, IGFBBP-3 and IGFBP-2. However, the effects were imprecisely estimates and some observed trends may justify larger trials.

## Abstract 2

Supplemental Digital Content is available in the text.

## Introduction

Prostate cancer (PCa) is a leading cause of morbidity and mortality among UK men (Cancer Research UK, 2016). Nutrition, in particular adopting a western lifestyle, is thought to be an important contributor to prostate carcinogenesis. The primary role of insulin-like growth factor (IGF) ligands (IGF-I, IGF-II) is to regulate prenatal and postnatal growth with binding proteins modulating their action (Daughaday and Rotwein, 1989; Jones and Clemmons, 1995). The IGF system also has an important role in carcinogenesis through proliferation, antiapoptotic and metabolic effects (Khandwala *et al.*, 2000) and high serum levels of IGF-I have been associated with risk of various cancers, including PCa (Yu *et al.*, 1999; Rowlands *et al.*, 2009; Young *et al.*, 2012; Rowlands *et al.*, 2013). In turn, nutrition has an important role in regulating the IGF system. It has, therefore, been hypothesized that dietary modification could reduce neoplastic growth and the risk of PCa by inhibiting both tumour initiation and growth of small cancer foci via IGF-related pathways. Laboratory and observational studies indicate that both lycopene and green tea may possess this dual action on cancer initiation and growth, but robust evidence from randomized controlled trials is needed to determine their causal effects in free living humans (Syed *et al.*, 2007).

Lycopene is a carotenoid found in tomatoes and tomato-based products (Najm and Lie, 2008). In-vitro and in-vivo studies suggest that lycopene is an antioxidant with potential anticancer effects (Wei and Giovannucci, 2012), inhibiting the viability of various human cancer cell lines, including PCa (Teodoro *et al.*, 2012). Supporting the laboratory data, a meta-analysis of 17 epidemiological studies indicated inverse associations of tomato or lycopene intake, and serum lycopene levels, with PCa (Jinyao Chen and Zhang, 2012). For nearly two decades studies have investigated associations between one or more carotenoids and the IGF axis. A recent cross-sectional study of 2742 men and 3316 women reported a 282 ng/ml [95% confidence interval (CI): 273–292, *P*_for trend_ = 0.01] and 245 ng/ml (95% CI: 237–253, *P*_for trend_ = 0.02) increase of IGF-I with the highest quintiles of lycopene in men and women, respectively. The ratio IGF-I: IGFBP-3 was also increased by 0.24 (95% CI: 0.23–0.25, *P*_for trend_ = 0.01) and 0.2 (95% CI: 0.19–0.21, *P*_for trend_ = 0.06) with highest quintiles of lycopene in men and women, respectively. There was also a trend that lycopene increases IGFBP-3 level but it was not conventionally statistically significant (Diener and Rohrmann, 2016).

It has been suggested that green tea and its active compound [epigallocatechin gallate (EGCG)], have numerous metabolic benefits (Ahmad *et al.*, 2015), including in-vitro and in-viv*o* evidence that they reduce the progression and invasion of numerous cancers, including PCa (Luo *et al.*, 2010; Fujiki *et al.*, 2015). These findings have preliminary support from a small, short-term randomized controlled trial in men with localized PCa, based on biochemical recurrence as the outcome (Thomas *et al.*, 2014), and a recent meta-analysis of 21 epidemiological studies (Fei *et al.*, 2014). Green tea has also been linked with the IGF axis in various cancers showing that EGCG counteracted IGF-I mediated angiogenesis in lung cancer cells (Li *et al.*, 2013) or was associated with reduction of IGF-I receptor in pancreatic cancer cells (Vu *et al.*, 2010). In-vivo studies also showed a decrease of IGF-I and restoration of IGFBP-3 levels after administering green tea polyphenol mixture (containing 62% of EGCG) to mice with transgenic adenocarcinoma of the prostate for 24 weeks (Adhami *et al.*, 2004).

To test the hypothesis that lycopene or green tea influence circulating IGF peptides in men at elevated risk of cancer (defined pragmatically in our study as men with prostate specific antigen (PSA) levels between 2.0 and 2.95 ng/ml or PSA ≥ 3 ng/ml but negative biopsies), we measured serum levels of IGF-I, IGF-II, IGF binding protein (BP)-2 and IGFBP-3 at baseline and after 6 months of an intervention in which men were randomized to daily lycopene and green tea (the ProDiet trial) (Lane *et al.*, 2010). Men adhered successfully to two dietary interventions with significant elevation of the primary outcomes, serum EGCG and lycopene levels at 6 months. Lycopene concentrations (µmol/l) were 25.4% higher (95% CI: 1.07–1.46, *P* = 0.005) in men following dietary advice and 41.9% higher (95% CI: 1.22–1.66, *P* < 0.001) following supplementation, both compared with placebo. Plasma EGCG levels in men assigned to dietary advice or green tea supplementation were raised by a median 22.1 nmol/l (95% CI: 2.59–41.61 nmol/l; *P* = 0.026) and 9.5 nmol/l (95% CI: −1.79–20.79 nmol/l; *P* = 0.099) versus placebo, respectively (Lane *et al.*, 2012).

## Patients and methods

The ProDiet trial was nested within the ProtecT (Prostate testing for Cancer and Treatment) trial (Lane *et al.*, 2010; Henning *et al.*, 2015) of treatments for localized PCa for men aged 50–69 years. The ProtecT men were recruited between 2001 and 2009 from 347 randomly selected general practices in the UK. In ProDiet, men with an increased risk of PCa (defined below) were assessed for their eligibility for entry into a double blind randomized controlled trial of dietary modifications with green tea and lycopene. The primary outcomes of this trial were feasibility of randomization, and the impact of the interventions on serum lycopene and EGCG levels after 6 months of follow-up as a measure of compliance to interventions. The IGF outcomes were exploratory (i.e. the prespecified power of the trial was not based on these outcomes).

Men were selected for inclusion in this trial from the 469 invited men at one ProtecT centre who had a PSA level between 2.0 and 2.95 ng/ml or a PSA of at least 3.0 ng/ml with a negative biopsy between 2008 and 2009. This group includes men with an elevated risk of PCa due to possible hidden precursor conditions (e.g. high-grade prostatic intraepithelial neoplasia) or small cancer foci in men with PSA below 3 ng/ml or that was undetected by biopsies. The following men were excluded: major comorbidities, other cancers or prior prostate malignancy; a PSA level of at least 20 ng/ml; a history of allergic reactions to green tea or lycopene containing products (including guava, watermelon); and current medication with finasteride or dutasteride (as these lower PSA levels). The men received both written and verbal information about the trial design and provided written informed consent. The Trent Multicentre Research Ethics Committee approved ProDiet (08/H0405/61).

### Randomization

The men were randomly assigned to receive one of three lycopene interventions – lycopene-rich diet: one or two daily portions of tomato-based foods or dishes (*n* = 44), lycopene capsules: one daily soft gel capsule of 15 mg tomato-derived lycopene (Lyc-*O*-mato, Lycored Ltd, Beer Sheva, Israel) (*n* = 44) or matched placebo capsule (provided by Lycored Ltd) (*n* = 45) – and one of the green tea interventions – green tea drink: two mugs or three cups of green tea daily (*n* = 45), green tea-derived capsules (Frutarom Ltd, Reinach, Switzerland) at 600 mg daily (*n* = 45), or matched placebo capsules (provided by Frutarom Ltd) (*n* = 43). Participants were provided with green tea (teabags and capsules) and lycopene capsules or placebos at randomization and at months 1 and 3 into the intervention. To aid compliance, a weekly record sheet was also provided for participants. In the lycopene intervention arm, men were also informed that cooking tomatoes increases lycopene’s bioavailability and that other sources of lycopene, such as watermelon or pink grapefruit, have a lower lycopene content compared with tomatoes.

### Food frequency questionnaire

Men filled in a validated 89 item self-completed food frequency questionnaire (FFQ) to record their average food intake over the 6 months before the trial (baseline assessment). Estimated daily intake of nutrients was calculated using a combination of estimated portion size from the FFQ and published composition of food data (McCance and Great Bristain, 2004). Any participants with at least 20 items missing from the FFQ had all nutrients recoded to missing, as previous research indicates that a missing response does not equate to ‘never’ in FFQs (Henning *et al.*, 2015). We used these data to derive intakes of: calcium (mg), animal protein (g), total protein (g) and total carbohydrates (g).

### Measurement of other baseline characteristics

All participants were asked to complete a questionnaire on family history of PCa, self-reported diabetes and sociodemographic status. BMI was calculated from self-reported height and weight as weight over height squared (kg/m^2^). Alcohol intake was estimated from the number of wine, beer or spirits units consumed and the amount of alcohol (g) per drink was calculated and reported as number of units of total alcohol per week. Smoking was categorized as: never, ever and current smokers. PSA was measured in the local PSA testing clinic at recruitment and at 6 months.

### Specimen collection and insulin-like growth factor-system assays

Nonfasted blood samples for IGF and IGFBPs measures were taken from men at their first appointment before diagnosis (baseline) and at the 6 months postrandomization follow-up appointment. The samples were allowed to clot, centrifuged at 1640*g* for 20 min within 2 h of collection and stored in −80°C until assayed. The samples underwent two freeze-thaw cycles before the IGF assays, which has previously been shown to have no effect on IGF or IGFBP-3 levels (Yu *et al.*, 1999). Circulating IGF-I, IGF-II (free and bound peptides) and IGFBP-3 (including all forms that had undergone minor fragmentations) levels were assessed using in-house radioimmunoassays by technicians blinded to randomization status: results used in the analysis were based on the mean of three measures. IGFBP-2 was assessed using an enzyme-linked immunosorbent assay (DY674; R&D Systems, Abingdon, UK); IGFBP-2 results used in the analysis were based on the mean of two measures. The average intra-assay coefficients of variation for IGF-I, IGF-II, IGFBP-3 and IGFBP-2 were 7, 9.5, 6.3 and 0.7%, respectively. The overall interassay coefficients of variation were 9.9, 11.3, 11.4 and 2.85%, respectively.

### Statistical analysis

We performed all statistical analysis in STATA version 2013 (StataCorp LLC, Collage Station, Texas, USA). Serum IGF-I, IGF-II and IGFBP-3 levels were approximately normally distributed. Serum IGFBP-2 levels were slightly positively skewed, but residuals were normally distributed with no major outliers, and we, therefore, analysed the raw data rather than perform a transformation that would be more difficult to interpret. We used linear regression to estimate associations of the interventions with follow-up IGFs and IGFBPs. There were no baseline imbalances in the covariates apart from diabetes status in green tea arm and so the primary analyses are unadjusted. We used analysis of variance to assess heterogeneity. In a secondary analysis, we controlled for measured baseline variables and in a sensitivity analysis we checked whether excluding men with diabetes made any difference to the results. We tested for interaction between the lycopene and green tea interventions on IGFs and IGFBPs using likelihood ratio tests.

## Results

**Fig. 1:**
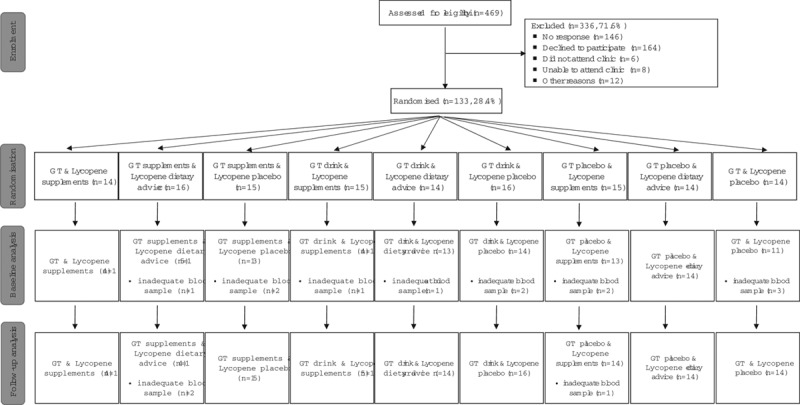
Flow diagram of study participants. GT, green tea.

At baseline, 120 men had IGFs measured, the missing data being due to inadequate serum sample collected. The 120 men were allocated to: (i) green tea – either as a green tea supplement (*n* = 41), green tea drink (*n* = 41) or placebo (*n* = 38); and (ii) lycopene, either as a lycopene supplement (*n* = 41), a lycopene-rich diet (*n* = 42) or placebo (*n* = 37). After 6 months of intervention, 130 men had follow-up IGFs measured: green tea supplement (*n* = 45), green tea drink (*n* = 44) or placebo (*n* = 41) and lycopene supplement (*n* = 41), lycopene-rich diet (*n* = 44) or placebo (*n* = 45) (Fig. 1).

Potential confounders (age, BMI and PSA) and IGF levels at baseline were mostly equally distributed between the lycopene and green tea (Table 1) intervention arms, confirming the success of randomization. We have also examined the potential for confounding due to family history of PCa, social class, smoking, alcohol and diabetes: all these variables were equally distributed between the groups and their inclusion in the models did not alter the results (data not shown). Only two men had diabetes and excluding them made no material difference to the results (Supplementary Table 1, Supplemental digital content 1, *http://links.lww.com/EJCP/A225*).

**Table 1:**
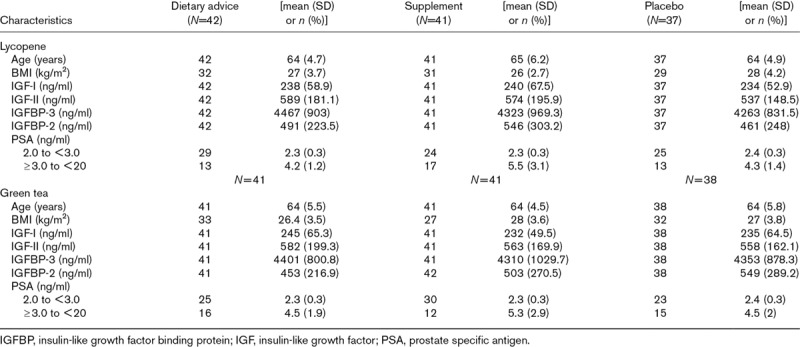
Distribution of each of the covariables and insulin-like growth factors in lycopene and green tea trial arms at baseline (120 men at risk of prostate cancer)

Table 2 shows there was little statistical evidence of any effect of the lycopene intervention (as a dietary modification or supplement vs. placebo) or green tea intervention (as a green tea drink or supplementation vs. placebo) on IGF-I, IGF-II, IGFBP-3 or IGFBP-2. In men randomized to lycopene supplements, IGFBP-2 was nonsignificantly 50.9 ng/ml (95% CI: −51.2–152.9, *P* = 0.3) higher in comparison to placebo, and in men randomized to green tea supplements, IGFBP-3 was nonsignificantly 205.2 ng/ml (95% CI: −583.3–172.9, *P* = 0.3) lower than with placebo. There was no evidence of interaction between the lycopene and green tea interventions on any of the IGF peptides (all *P*_for interaction_ > 0.25).

**Table 2:**
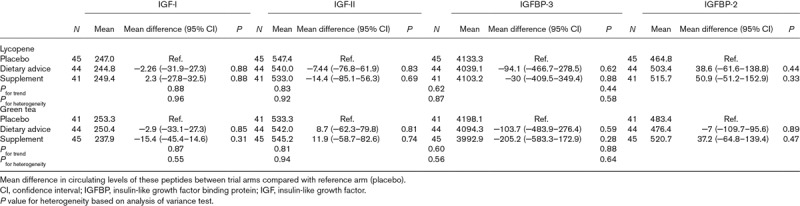
Mean (ng/ml) and mean differences (95% confidence interval) in insulin-like growth factors and insulin-like growth factor binding proteins according to lycopene or green tea intervention in 130 men at risk of prostate cancer (unadjusted)

## Discussion

### Main finding

In this small, pilot randomized controlled trial, there was no statistical evidence that increased tomato intake, lycopene supplementation, green tea drink or green tea supplementation influenced IGF-I, IGF-II or IGFBP-3 and IGFBP-2. However, the sample size in this pilot was small and thus, effect estimates were imprecisely estimated (wide CIs). There was suggestive evidence that in men randomized to lycopene supplements, IGFBP-2 was higher in comparison to placebo, and in men randomized to green tea supplements, IGFBP-3 was lower than with placebo.

### Comparison with the literature

A meta-analysis of 11 239 cases, suggested that the risk of aggressive PCa in men within the highest versus lowest quintile of lycopene intake was 0.65 (95% CI: 0.46–0.91) (Key *et al.*, 2015). However, a more recent post-hoc analysis from the Procomb trial did not show evidence for or against selenium and lycopene supplementation with PCa patients (Morgia *et al.*, 2017). In the Procomb trial, daily lycopene intake was given at a dose of 5 mg for a year compared with 15 mg of lycopene daily in our trial. In another meta-analysis, consumption of green tea was found to have a protective effect on risk of PCa (odds ratio=0.43, 95% CI: 0.25–0.73) (Zheng *et al.*, 2011). Drinking green tea (six cups per day) was also associated with a reduction in PSA levels (Henning *et al.*, 2015).

In-keeping with our findings, an open-label study of 26 men with PCa showed that short-term (6 weeks on average) supplementation with EGCG (800 mg daily) resulted in a reduction in IGF-I serum level (SD) by 28 ng/ml (57.2), IGFBP-3 by 291 ng/ml (606) and IGF-I: IGFBP-3 ratio by 0.0029 (0.0123) (McLarty *et al.*, 2009). Our study and others are relatively small in size and may justify larger trials to verify that they are not simply chance.

In relation to the lycopene intervention, there was little evidence of an association with IGF-I, IGF-II or IGFBP-3 levels, but weak, imprecise evidence of an effect on IGFBP-2. Other studies showed no effect of lycopene on IGF-I, IGF-II or IGFBP-3 in patients with colorectal cancer (Vrieling *et al.*, 2007), no effect on IGF-I in breast cancer patients (Voskuil *et al.*, 2008) and no effects on IGF-I in healthy participants (Riso *et al.*, 2006). A small study of 58 men with high-grade prostatic intraepithelial neoplasia taking lycopene (30 mg/day) over the same period (6 months) as this study, found no changes in serum IGF-I (*P* = 0.99) and IGFBP-3 (*P* = 0.53) levels between pretreatment and post-treatment groups (Gann *et al.*, 2015). Another study showed no effect on IGF-I (*P* = 0.93) and IGF-I receptor (*P* = 0.53) in group of 22 men with favourable risk of PCa when on 3 months intervention with lycopene (30 mg/day) versus placebo (Chan *et al.*, 2011). Men and women with colorectal cancer, randomly assigned to lycopene capsules for 8 weeks had increased levels of serum IGFBP-2 (by 8.2%, 95% CI: 0.7–15.6% for men and 7.8%, 95% CI: −5.0–20.6% for women). This increase in circulating IGFBP-2 could potentially reduce IGF-I bioavailability (Gann *et al.*, 2015). However, in our study there was no clear statistical evidence that the lycopene intervention impacted IGFBP-2.

There are numerous observational studies in relation to nutrition and PCa but many of them are likely to be confounded by diet itself, physical activity and other common causes of both nutritional exposures and PCa. A meta-analysis of observational studies showed a pooled odds ratio estimate for PCa in the highest versus non/lowest green tea consumption of 0.72 (95% CI: 0.45–1.15) (Zheng *et al.*, 2011). Another meta-analysis indicated a beneficial effect of tomatoes (but not lycopene) in relation to risk of PCa with pooled risk estimates of 0.81 (95% CI: 0.69–1.06, *P* = 0.09) and 0.97 (95% CI: 0.88–1.08, *P* = 0.52), respectively (Jinyao Chen and Zhang, 2012). However, we cannot speculate that green tea or lycopene intervention modify the risk of PCa via the IGF system based on our results.

The major strength of our study is the random assignment, and baseline IGFs, IGFBPs and covariates were equally distributed among trial arms suggesting successful randomization. Another strength was the good adherence to the intervention as evidenced by the serum levels of lycopene and EGCC plus the effect of supplementation was assessed with both capsular and dietary options. Detection bias was excluded as all staff was blinded to randomization. The major weakness is that the trial was not set up to investigate IGFs as a primary outcome and was designed as a feasibility pilot study. Therefore, we may not have been able to detect important effects that may be seen in adequately powered trials. Hence, our findings are exploratory.

### Conclusion

In this small pilot, randomized controlled trial, there was no clear evidence that tomato-enriched diet, lycopene supplementation, or a green tea intervention (drink or tablets) influenced IGF-I, IGF-II, IGFBP-3 or IGFBP-2 serum levels.

## Acknowledgements

The authors acknowledge the tremendous contribution of all members of ProtecT study research group and men who participated in ProDiet study.

This study was funded by Cancer Research UK (C11046/A10052) and by the National Institute for Health Research (NIHR) Bristol Nutritional Biomedical Research Unit based at University Hospitals Bristol NHS Foundation Trust and the University of Bristol. This study was designed and delivered in collaboration with the Bristol Randomised Trials Collaboration (BRTC), a UKCRC registered clinical trials unit which, as part of the Bristol Trials Centre, is in receipt of National Institute for Health Research CTU support funding. The views expressed are those of the authors and not necessarily those of the NHS, the NIHR or the Department of Health. R.M.M., J.M.P.H. and J.A.L. are supported by a Cancer Research UK (C18281/A19169) Programme Grant (the Integrative Cancer Epidemiology Programme). C.J.B. was funded by a Welcome Trust 4-year PhD studentship (WT083431MA) and Diabetes UK (17/0005587).

The ProtecT study is funded by the UK Health Technology Assessment (HTA) Programme of the National Institute for Health Research HTA 96/20/00; ISRCTN20141297. The authors would like to acknowledge the support the National Cancer Research Institute (NCRI) formed by the Department of Health, the Medical Research Council (MRC) and Cancer Research UK. The NCRI provided founding through ProMPT (Prostate Mechanisms of Progression and Treatment), and this support is gratefully acknowledged. D.G., J.D., F.H. and D.N. are NIHR Senior Investigators. The funders had no role in study design, data collection an analysis, decision to publish or preparation of the manuscript.

## Conflicts of interest

There are no conflicts of interest.

## Supplementary Material
