# Supplementary material for: Effect of green tea and lycopene on the insulin-like growth factor system: the ProDiet randomized controlled trial
Source: Eur J Cancer Prev. 2018 Mar 15;28(6):569–75. doi: 10.1097/CEJ.0000000000000502 (PMC6784856; doi:10.1097/CEJ.0000000000000502)
Supplement: Supplementary file 1 [file ejcp-28-569-s001.docx]

**Supplementary Table 1.**

Means (ng/ml) and mean differences (95% CI) in IGFs and IGFBPs according to lycopene or green tea intervention in 128 men at risk of prostate cancer (excluding men with diabetes)

|  | **IGF-I** | | | | | **IGF-II** | | | | **IGFBP-3** | | | | | | **IGFBP-2** | | |
| --- | --- | --- | --- | --- | --- | --- | --- | --- | --- | --- | --- | --- | --- | --- | --- | --- | --- | --- |
|  |  | **Mean** | **Mean diff.**  **(95% CI)** | **p** |  | | **Mean** | **Mean diff.**  **(95% CI)** | **p** |  | **Mean** | **Mean diff.**  **(95% CI)** | **p** |  | **Mean** | | **Mean diff.**  **(95% CI)** | **p** |
| **Lycopene** |  |  | |  |  | |  | |  |  |  | |  |  |  | | |  |
| **Placebo** | 45 | 247.0 | (ref) |  | 45 | | 547.4 | (ref) |  | 45 | 4133.3 | (ref) |  | 45 | 464.8 | | (ref) |  |
| **Dietary advice** | 44 | 244.8 | -2.3 (-31.9; 27.3) | 0.88 | 44 | | 540.0 | -7.4 (-76.8; 61.9) | 0.83 | 44 | 4039.1 | -94.1 (-467.6; 279.4) | 0.62 | 44 | 503.4 | | 38.6 (-61.8; 138.9) | 0.45 |
| **Supplement** | 39 | 252.7 | 5.7 (-24.9; 36.2) | 0.71 | 39 | | 540.5 | -6.9 (-78.5; 64.6) | 0.85 | 39 | 4124.3 | -8.9 (-394.4; 376.4) | 0.96 | 39 | 525.7 | | 60.9 (-42.7; 164.5) | 0.25 |
| p for trend |  |  | | 0.88 |  | |  | | 0.83 |  |  | | 0.62 |  |  | | | 0.44 |
| p for heterogeneity |  |  | | 0.87 |  | |  | | 0.97 |  |  | | 0.86 |  |  | | | 0.49 |
|  |  |  | |  |  | |  | |  |  |  | |  |  |  | | |  |
| **Green tea** |  |  | |  |  | |  | |  |  |  | |  |  |  | | |  |
| **Placebo** | 41 | 253.3 | (ref) |  | 41 | | 533.3 | (ref) |  | 41 | 4198.1 | (ref) |  | 41 | 483.4 | | (ref) |  |
| **Dietary advice** | 44 | 250.4 | -2.9 (-33.2; 27.4) | 0.85 | 44 | | 542.0 | 8.7 (-62.2; 79.7) | 0.81 | 44 | 4094.3 | -103.7 (-485.1; 277.7) | 0.59 | 44 | 476.4 | | -7 (-109.9; 95.8) | 0.89 |
| **Supplement** | 43 | 240.4 | -12.9 (-43.4; 17.5) | 0.40 | 43 | | 552.6 | 19.3 (-52.1; 90.6) | 0.59 | 43 | 4006.8 | -191.2 (-574.7; 192.3) | 0.33 | 43 | 530.0 | | 46.6 (-56.8; 150) | 0.37 |
| p for trend |  |  | | 0.86 |  | |  | | 0.81 |  |  | | 0.60 |  |  | | | 0.87 |
| p for heterogeneity |  |  | | 0.67 |  | |  | | 0.87 |  |  | | 0.61 |  |  | | | 0.53 |
